# Survivin expression in canine spontaneous cutaneous and subcutaneous tumors and its prognostic importance

**DOI:** 10.14202/vetworld.2017.1286-1291

**Published:** 2017-10-30

**Authors:** N. Kavya, S. Rao, M. L. Sathyanarayana, H. D. Narayanaswamy, S. M. Byregowda, L. Ranganath, A. Kamaran, K. M. Purushotham, T. K. Kishore

**Affiliations:** 1 Department of Veterinary Pathology, Veterinary College, Karnataka Veterinary Animal and Fisheries Sciences University, Bengaluru, Karnataka, India; 2 Department of Biotechnology, Institute of Animal Health and Veterinary Biologicals, Veterinary College, Karnataka Veterinary Animal and Fisheries Sciences University, Bengaluru, Karnataka, India; 3 Department of Veterinary Surgery and Radiology, Veterinary College, Karnataka Veterinary Animal and Fisheries Sciences University, Bengaluru, Karnataka, India; 4 Department of Veterinary Medicine, Veterinary College, Karnataka Veterinary Animal and Fisheries Sciences University, Bengaluru, Karnataka, India

**Keywords:** cutaneous and subcutaneous tumors, prognostic value, quantitative real-time polymerase chain reaction, survivin

## Abstract

**Aim:**

The present study was carried out to know the expression level of survivin, an inhibitor of apoptosis protein with an objective to determine its prognostic importance in cutaneous and subcutaneous tissue tumors of dogs.

**Materials and Methods:**

Forty cases of canine cutaneous and subcutaneous tissue tumors on histopathological examination revealed various round cell, epithelial, and mesenchymal cell tumors. Survivin gene expression was detected in all tumors tested by TaqMan real-time polymerase chain reaction assay by comparative cycle threshold method.

**Results:**

The mean survivin gene expression value of benign tumors was 0.94±0.63 folds and that of malignant tumors was 18.87±5.30 folds. Postsurgical follow up of 30 malignant tumor cases revealed death in 8, recurrence in 7, and neoplastic free alive status in 15 dogs with mean survivin fold difference values of 48.49±12.39, 14.63±6.37, and 5.034±2.27, respectively. The mean survivin gene expression value was significantly higher in malignant (30 cases, 18.87±5.30) compared to benign tumors (10 cases, 0.94±0.63), and it varied between various postsurgical follow-up groups (p<0.05). Survival analysis, using survivin gene expression median cutoff value of 3.74 in 30 malignant tumors, was performed to predict probable survival period in malignant cutaneous and subcutaneous tumors of dogs.

**Conclusions:**

Results of the present study indicated that the expression of survivin in canine cutaneous and subcutaneous tumors has prognostic value, and survivin expression greater than median cutoff value of 3.74 has a poor prognosis.

## Introduction

Cancer is a consequence of imbalance between cell death and proliferation in a way favorable to cell proliferation and survival [1]. In the recent years, cancer has been reported to be a leading cause of mortality in dogs and second most in humans [2]. Cutaneous and subcutaneous tissue tumors account for approximately one-third of all the tumors encountered in dogs [3,4] with most common types being epithelial tumors, followed by round cell tumors and mesenchymal tumors [5-7]. The peak age of affection in dogs is 8-10 years [8], and breed and sex of dogs have no significant influence on the incidence of skin tumors [7,9,10]. Approximately 20-40% of primary tumors of the skin and subcutaneous tissues are histologically malignant in the dogs [7] and have a higher tendency of recurrence and metastasis to visceral organs, resulting in reduced survival time and rate of affected dogs [11,12]. Hence, accurate diagnosis of malignancy at the earliest stage is one of the requirements for effective management of cancers. Recent advances in tumor biology have identified a number of markers that may form a basis for tumor diagnosis and prognosis [13].

Inhibitors of apoptosis proteins (IAPs) are a family of tumor markers that interfere with the activation of caspases [14]. Survivin, one of the members of IAP is a bifunctional protein that regulates cell division and suppresses apoptosis [15]. Although survivin is abundantly expressed in fetal tissues, it is undetectable in most normal, terminally differentiated adult tissues [16]. It is overexpressed in a variety of human neoplasms including breast [17,18], esophagus [19], stomach [20,21], colon [22], pancreas [23], bladder [24], renal cell [25], head and neck [26], oral [27], and leukemias [28], suggesting that reactivation of the survivin gene frequently occurs in cancers [29].

High survivin expression by neoplasms correlates with more aggressive behavior, decreased response to chemotherapeutic agents, and shortened survival time as compared to cancers that are survivin negative [29]. Intensive survivin research is currently ongoing in human field, which is being used as a prognostic factor in several human neoplasms. Its expression in companion animals has limited investigation, and hence, the present study was carried out to know the expression level of survivin with an objective to determine its prognostic importance in cutaneous and subcutaneous tissue tumors of dogs.

## Materials and Methods

### Ethical approval

Ethical approval is not necessary for such type of clinical cases. However, surgery was conducted upon consent from owner and as per standard surgical methods.

### Place of study

This study was conducted in the Department of Veterinary Pathology, Veterinary College, Hebbal, Bengaluru, during 2015-2016, on 40 cases of cutaneous and subcutaneous tissue tumors of dogs presented to the Department of Veterinary Surgery, Veterinary College, Bengaluru.

### Study animals and sample collection

Breed of dogs encountered in the present study was mongrels (13), followed by Labrador Retriever (8), Boxer (5), German Shepherd (4), Pomeranian (3), Golden Retriever (2), and Doberman (2). The other breeds which were affected lesser were Pug, Dalmatian, and Rottweiler. Apart from visible skin masses, patients were declared as healthy based on pre-operative hematological and serological parameters. On owner’s consent, surgery was carried out for cutaneous masses, and tissues were collected for histopathological processing. A follow-up study for a minimum period of 8 months was carried out postsurgically in all the dogs.

### Histopathology

Representative tissue samples obtained after surgical excision were fixed in 10% neutral buffered formalin and processed by routine paraffin-embedding technique. Sections of 4-5 µm thickness were taken, and cut sections were stained with hematoxyline and eosin. Tissue sections were examined to record and classify the cutaneous and subcutaneous tumors.

### Quantitative real-time (q-RT)-polymerase chain reaction (PCR) for survivin mRNA expression

Total RNA was isolated using TRIzol^®^ ready to use solution procured from M/s Invitrogen (USA) and used as per manufacturer’s recommendations. Complementary DNA (catalog no. 3B 120, biotools B and M Labs, Spain) was prepared for RNA sequences encoding survivin gene of dog using gene-specific primers. Quantitative TaqMan RT-PCR (catalog no. 3B 108, biotools B and M Labs, Spain) assay was carried out for survivin (antiapoptotic protein) and glyceraldehyde-3-phosphate dehydrogenase (GAPDH) (housekeeping gene) mRNA using the Thermal cycler (EPPENDORF realplex 2.2) instrument according to the manufacturer’s instructions. Published sequences available in the gene bank were used for the designing of required primers for the study. Primers were designed using primer blast (http://www.ncbi.nlm.nih.gov/tools/primerblast/), Genscript^®^ (https://www.genscript.com/sslbin/app/primer), and primer3plus^®^ (http://www.bioinformatics.nl/cgibin/primer3plus/primer3plus.cgi/) sequence analyzing software’s and procured from Bioserve Biotechnologies (India) Pvt Ltd.

The published reference sequence of survivin for dog was from NCBI No: NM-001003348 (Table-1).

**Table-1:** Primers and probes used for q-RT-PCR.

Primer code		Primer sequence	Product size (bp)
Canine survivin F	5’-3’	TCATCTGGTTGTGCTTTCCT	88
Canine survivin R	5’-3’	TGGCTCTTTCTTTGTCCAGT	
Survivin probe	3’-5’	TCTGTCAAGAAGCAGTTTGAAGA	
GAPDH F	5’-3’	ATGACTCTACCCACGGCAAG	106
GAPDH R	5’-3’	TACTCAGCACCAGCATCACC	
GAPDH probe	5’-3’	AAACCCATCACCATCTTCCAG	

q-RT-PCR=Quantitative real-time-polymerase chain reaction, GAPDH=Glyceraldehyde-3-phosphate dehydrogenase

RT-PCR amplification reaction was carried out in a 20 µl reaction mixture containing 10 µl each of mastermix (3B quantimix), and samples were used in duplicate. Relative gene quantification was done by comparative Ct method, and the values were expressed as relative to the reference sample used, as calibrator (Tables-2 and 3).

**Table-2:** Thermal cycling conditions for amplification of dog GAPDH gene.

Stage	Temperature (°C)	Duration	Number of cycles
Initial denaturation	95	3 min	1
Denaturation of cDNA	95	5 s	40
Annealing of primers	59.6	20 s	
Extension	65	20 s	

GAPDH=Glyceraldehyde-3-phosphate dehydrogenase

**Table-3:** Thermal cycling conditions for amplification of dog survivin gene.

Stage	Temperature (°C)	Duration	Number of cycles
Initial denaturation	95 s	3 min	1
Denaturation of cDNA	95	5 s	40
Annealing of primers	52	20 s	
Extension	65	20 s	

### Statistical analysis

Statistical analysis was performed using the statistical software R version 3.2.4 Revised Copyright (C) 2016 (R-bloggers.com). Mean values and standard error of the mean were calculated, and all values were expressed as mean±standard error. The data were analyzed by t-test unpaired, ANOVA-Tukey test was used for finding the source of the differences in multiple groups, and Kaplan–Meier survival curve analysis and curve were compared by log-rank test. For all statistical analysis, p<0.05 was considered statistically significant.

## Results

**Figure-1:**
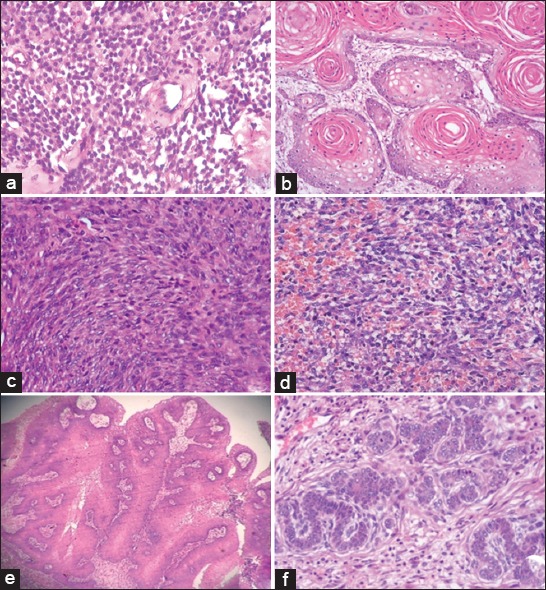
Histopathological images of various cutaneous and subcutaneous tumors H and E. (a) Mast cell tumor, (b) squamous cell carcinoma, (c) fibrosarcoma, (d) hemangiosarcoma, (e) squamous papilloma, and (f) trichoblastoma.

In this study, 40 cutaneous and subcutaneous tumors were classified based on the predominant cell type and histological characteristics (Figure-1) as round cell tumors (8 cases, 20%), epithelial tumors (23 cases, 57.5%), and mesenchymal tumors (9 cases, 22.5%). Out of 40 cases, 30 (75%) were malignant and remaining 10 (25%) were benign tumors. The malignant tumors predominated over the benign types in the present study, and epithelial tumors predominated over other types.

All the 40 tumor cases were subjected for immunohistochemistry to know the expression profile of survivin using EP 119 rabbit monoclonal primary antibody raised against human survivin protein which was procured from PathnSitu, Bengaluru. In positive control sections of human breast cancer tissues, the EP 119 monoclonal antibody reacted well and detected the survivin antigen (PathnSitu, Bengaluru). However, the application of IHC technique using anti-survivin EP119 antibody on normal and tumorous canine cutaneous and subcutaneous tissue did not yield any reaction on repeated testing with modifications in antigen retrieval methods and immunostaining incubation period as well as incubation temperature. Hence, the survivin gene expression to determine its prognostic value in the present study was carried out by RT-PCR on tumor tissues for its relative quantification.

Relative quantification of survivin gene was carried out to determine the level of expression of survivin gene in malignant and benign cutaneous and subcutaneous tumor tissues and normal canine skin tissues as control. The survivin gene expression in malignant tumors varied from 1.21 to 95.01 folds, and among benign tumors, it was 0.01-8.4 folds. The mean survivin gene expression of benign tumors was 0.94±0.63 folds and that of malignant tumors was 18.87±5.30 folds. Among all the malignant tumors, highest mean survivin gene expression value was observed in sweat gland adenocarcinoma (65.26±29.75), and the least expression was in malignant trichoepithelioma (1.96±0.75) (Table-4). Statistical analysis revealed that the mean expression of survivin gene was significantly higher (p<0.05) in malignant tumors.

**Table-4:** Mean±SE of survivin gene expression values of different cutaneous and subcutaneous tumors (n=40).

Type of tumor	Number of cases	Mean±SE survivin gene expression
Round cell tumors (n=8)		
Malignant type		
Mast cell tumor	4	25.57±20.74
Histiocytoma	3	2.23±0.48
Transmissible venereal tumor	1	5.28
Epithelial tumors (n=23)		
Benign type (n=7)		
Fibropapilloma	1	0.01
Squamous papilloma	1	0.03
Benign trichoblastoma	2	5.1±3.3
Acanthomatous ameloblastoma (Epulis)	1	0.3
Pilomatricoma	2	0.015±0.01
Malignant type (n=16)		
Squamous cell carcinoma	5	22.02±15.88
Malignant trichoepithelioma	2	1.96±0.75
Hepatoid gland adenocarcinoma	3	17.04±15.4
Sweat gland carcinoma	2	65.26±29.75
Solid adenocarcinoma of mammary gland	2	38.33±24.7
Complex adenocarcinoma of mammary gland	1	10.93
Simple adenocarcinoma of mammary gland	1s	23.59
Mesenchymal tumors (n=9)		
Benign type (n=3)		
Lipoma	1	0.27
Hemangioma	1	1.81
Fibroma	1	0.01
Malignant type (n=6)		
Fibrosarcoma	3	4.39±2.01
Hemangiosarcoma	3	9.86±6.4

SE=Standard error

To determine the prognostic value of survivin gene expression, a follow-up study was conducted for 30 malignant cutaneous and subcutaneous tumors for a minimum period of 8 months. There were deaths in 8 cases, recurrence in 7 cases, and disease-free status in 15 cases postsurgically. The median cutoff value of survivin gene expression for 30 malignant cutaneous and subcutaneous tumors tissues was 3.74. The tumors bearing expression value more than median cutoff were considered as overexpressed and those with less than median value as underexpressed. Among 30 malignant tumors, 15 (50%) cases showed overexpression, and 15 (50%) showed underexpression (Table-5). There was overexpression of survivin gene in all the 8 cases that died and the tumors encountered were mammary tumors, sweat gland tumor, and mesenchymal tumors such as fibrosarcoma and hemangiosarcoma. Among the 7 cases that showed recurrence, survivin was overexpressed in 4 cases with squamous cell carcinoma being the most common tumor, and rest of the 3 cases showed underexpression. In the alive group of tumor cases, 12 showed underexpression of survivin and three showed overexpression.

**Table-5:** Subdivision of various postsurgical outcome groups using median cutoff value of survivin gene expression (3.74).

Survivin expression	Alive (%)	Dead (%)	Recurrence (%)
Survivin expression<3.74 (underexpression)	12 (80)	0 (0)	3 (42.86)
Survivin expression>3.74 (overexpression)	3 (20)	8 (100)	4 (57.14)

**Figure-2:**
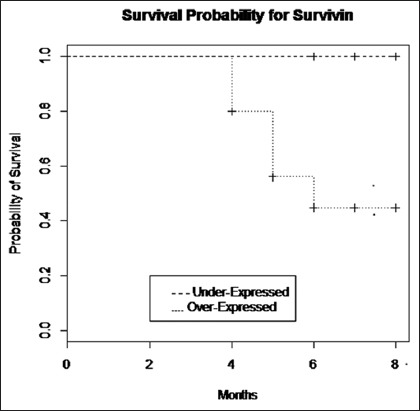
Kaplan–Meier survival curves for dogs with malignant tumors having survivin gene expression more than and less than the calculated median value of 3.74 (p≤0.05).

A statistically significant (p<0.05) difference in expression of survivin was observed between dead and disease-free alive groups. However, no significant difference was observed between alive and recurrence groups (Table-6). In assessing long-term results of the surgical treatment using Kaplan–Meier survival curves of follow-up period of dogs revealed statistically significant (p≤0.05) difference in survival time between dogs having survivin gene expression values above and below the median cutoff value of 3.74. At 0.5 level of probability, the mean survival time for dogs with survivin gene expression values more than the median value was found to be 6 months and less than the median value was undefined (Figure-2).

**Table-6:** Mean±SE of survivin gene expression in various postsurgical outcome groups.

Follow-up data	Total number of cases and percentage	Mean±SE of survivin gene expression
Alive	15 (50)	5.03±2.27^a^
Recurrence	7 (23.33)	14.63±6.37^a^
Dead	8 (26.67)	48.49±12.39^b^

Means bearing different superscripts are significantly different at P<0.05, SE=Standard error

## Discussion

Canine survivin is 91.5% homologous to its human counterpart at the amino acid level [30]. The expression level of survivin gene depends on several variables such as degree of differentiation of tumor, histologic grade, mitotic index, and type of tumor. The gene expression is lower in well-differentiated tumors with histologic grade zero, and mature cells do not show survivin expression [31,32]. However, reactivation of the survivin gene frequently occurs in neoplastic processes.

The probable reason for failure to detect survivin in normal and tumorous canine cutaneous and subcutaneous tissue by immunohistochemistry could be due to the lack of cross-reactivity of human anti-survivin monoclonal antibody to canine survivin antigen.

The survivin gene expression study indicated that the malignant tumors express a higher level of survivin than benign types and have diagnostic and prognostic importance.

High survivin expression by neoplasms correlates with more aggressive behavior and shortened survival time as compared to cancers that are survivin negative [29]. All eight dogs that died during postsurgical follow-up period revealed overexpression of survivin gene which indicated the association between malignancy and survivin expression and its prognostic significance. Several earlier workers also have indicated the prognostic importance of survivin in various malignant neoplasms of humans and animals [1,32-37]. The results of the study also indicating that there was no difference in the level of expression of survivin between recurrence and alive groups as some of the alive cases also revealed higher surviving expression.

Statistical analysis of Kaplan–Meier survival curves for dogs with under- and over-expression groups in relation to various postsurgical outcomes using median cutoff value of 3.74 was compared with log-rank test. Mean survival time for dogs with survivin expression more than the median value was observed to be shorter, indicating that survivin expression correlates with the postsurgical outcome, and the higher expression above the median value has a poor prognosis.

## Conclusion

It may be concluded that by setting median cutoff value for survivin gene expression (3.74 as in the present 40 cases of cutaneous and subcutaneous tumors), prognosis of tumor patients could be determined with relation to various postsurgical outcome groups. Survivin gene expression value lower than median cutoff value will have a more favorable prognosis and guarded prognosis when the value is higher than the median value.

However, the results from the current study need to be further validated in a large number of cases.

## Authors’ Contributions

NK: Carried out the research work. SR and NK: Sample collection, gross, histopathological work, photography, and interpretation of data, collection of scientific literatures, and preparation of first draft of the manuscript were done by these authors. SR, MLS, HDN, and AK: Supervised the work. RL: Contributed in sample collection. NK, SMB, and KMP: Contributed in RT-PCR. TKK: Helped in research work. All authors read and approved the final manuscript.
